# Water and soil pollution as determinant of water and food quality/contamination and its impact on male fertility

**DOI:** 10.1186/s12958-018-0449-4

**Published:** 2019-01-06

**Authors:** Andrea Di Nisio, Carlo Foresta

**Affiliations:** Department of Medicine, Unit of Andrology and Reproductive Medicine, University of Padova, Via Giustiniani, 2, 35128 Padova, Italy

**Keywords:** Endocrine disruptors, Infertility, Sexual development, Male health, Seminal parameters

## Abstract

Over the past two decades, public health has focused on the identification of environmental chemical factors that are able to adversely affect hormonal function, known as endocrine disruptors (EDs). EDs mimic naturally occurring hormones like estrogens and androgens which can in turn interfere with the endocrine system. As a consequence, EDs affect human reproduction as well as post and pre-natal development. In fact, infants can be affected already at prenatal level due to maternal exposure to EDs. In particular, great attention has been given to those chemicals, or their metabolites, that have estrogenic properties or antagonistic effects on the activity of androgen or even inhibiting their production. These compounds have therefore the potential of interfering with important physiological processes, such as masculinization, morphological development of the urogenital system and secondary sexual traits. Animal and in vitro studies have supported the conclusion that endocrine-disrupting chemicals affect the hormone-dependent pathways responsible for male gonadal development, either through direct interaction with hormone receptors or via epigenetic and cell-cycle regulatory modes of action. In human populations, epidemiological studies have reported an overall decline of male fertility and an increased incidence of diseases or congenital malformations of the male reproductive system. The majority of studies point towards an association between exposure to EDs and male and/or female reproductive system disorders, such as infertility, endometriosis, breast cancer, testicular cancer, poor sperm quality and/or function. Despite promising discoveries, a causal relationship between the reproductive disorders and exposure to specific toxicants has yet to be established, due to the complexity of the clinical protocols used, the degree of occupational or environmental exposure, the determination of the variables measured and the sample size of the subjects examined. Despite the lack of consistency in the results of so many studies investigating endocrine-disrupting properties of many different classes of chemicals, the overall conclusion points toward a positive association between exposure to EDs and reproductive system. Future studies should focus on a uniform systems to examine human populations with regard to the exposure to specific EDs and the direct effect on the reproductive system.

## Introduction

Endocrine disruptors (EDs) are exogenous chemical entities or mixtures of compounds that interfere with any aspect of hormone action responsible for the maintenance of homeostasis and the regulation of developmental processes. The research conducted in the field of EDs has increased considerably over the last two decades, due to their potentially adverse effects on human health, supported by increasing experimental evidence in the areas of developmental biology and environmental toxicology. More specifically, it is well known that chemicals interfering with hormonal pathways can seriously affect human reproduction. Several studies have demonstrated a significant decrease in fertility biomarkers, notably sperm counts, in human populations that have been exposed to EDs [1–4]. The toxic effects of EDs have resulted in the restriction of their use in countries where evidence of extensive exposure is wide [5]. In some westernized countries, the use of certain EDs has been banned. However, in some cases the human exposure to EDs is inevitable, when such chemicals are used in occupational activities or are widely dispersed across the environment. The daily used products like pesticides, plastic items containing bisphenol A and phthalates, flame retardants, personal care products containing antimicrobials, heavy metals and perfluoroalkyls are regularly being manufactured in the industries. These are some of the most potential candidates of endocrine disruptors. From these industries, chemicals are easily released into the environment for example through leaching into the soil and water. These are then taken up by microorganisms, algae and plants which are then taken up by animals. After this, endocrine disruptors find their way in the food chain from the animals to finally into human being [6].

Over the past two decades, public health has focused on the identification of environmental chemical factors that are able to adversely affect hormonal function [7]. EDs mimic naturally occurring hormones like estrogens and androgens which can in turn interfere with the endocrine system. EDs are highly heterogeneous and can be classified according to their origins in: i) Natural and artificial hormones (e.g. fitoestrogens, 3-omegafatty acids, contraceptive pills and thyroid medicines); (ii) drugs with hormonal side effects (e.g.naproxen, metoprololand clofibrate); (iii) industrial and household chemicals (e.g. phthalates, alkylphenoletoxilate detergents, plasticizers, solvents) and (iv) side products of industrial and household processes (e.g. polycyclic aromatic hydrocarbons, dioxins, pentachlorobenzene). Given their widespread diffusion and environmental exposure not only limited to professional activities, this review will focus only on the 3rd class of EDs.

**Fig. 1:**
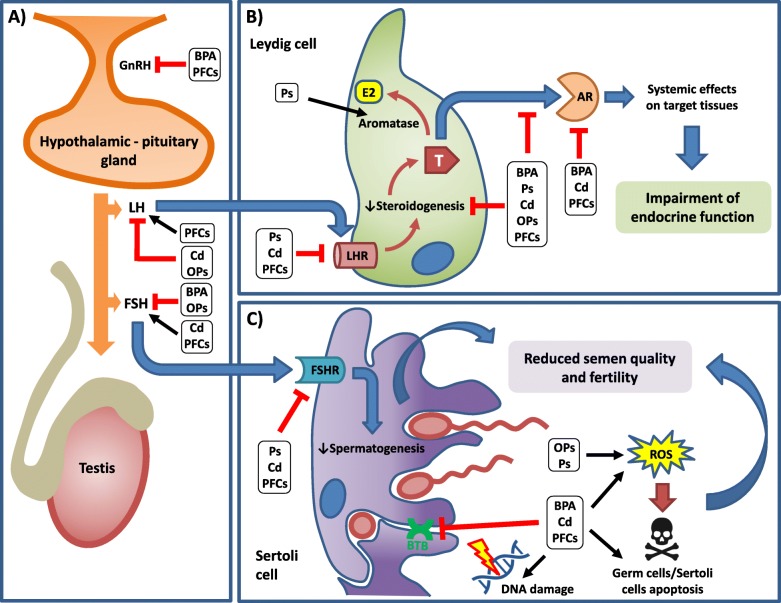
Schematic representation of endocrine disruptors’ (EDs) effects on male fertility and the related mechanisms of toxicity. Results from both pre-clinical and clinical studies are summarized for each ED. If proposed effects are common to more EDs, they are reported together within black squares. Black arrows refer to stimulatory pathways. Red arrows with blunt ends represent inhibitory regulation. Hypotalamic-pituitary regulation of testicular function is impaired by most EDs (**a**). Within the testis, gonadotropins stimulates steroidogenesis in Leydig cells (**b**) and spermatogenesis in Sertoli cells (**c**). Overall, EDs disrupt endocrine function by reducing testosterone release or its activity on target tissues. In addition, EDs can reduce semen quality by directly impairing cell structure/viability or indirectly by interfering with hormonal patwhays. GnRH: Gonadotropin-releasing hormone; LH: Lutehinizing Hormone; FSH: Follicle-Stimulating Hormone; T: testosterone; AR: Androgen Receptor; FSHR: FSH Receptor; LHR: LH Receptor; E2: Estradiol; ROS: Reactive Oxygen Species; BTB: Blood-Testis Barrier; BPA: Bisphenol A; Ps: Phtalathes; Cd: Cadmium; Ops: Organophosphate pesticides; PFCs: Perfluoroalkyl Compounds

EDs exert their toxicity by interfering with the normal hormonal homeostatic mechanisms that promote growth and development of tissues. The classical action with respect to the reproductive system involves interference of EDs with hormone binding to the corresponding receptor, notably the androgen receptor (AR) or the estrogen receptor (ER). Following binding to a receptor the ED can trigger two types of responses: a hormonal response that is termed an agonistic effect, and/or a lack of hormonal response that is termed an antagonistic action. In addition to the hormone-related receptors, EDs act on enzymes involved in steroidogenesis and the metabolism of hormones [8] (Fig. 1).

The effects of EDs on the male reproductive system are notably attributed to the interactions of these chemicals with the normal production and/or function of steroid hormones that are responsible for the initiation of prostate development and the masculinization of the Wolffian ducts in order to form the epididymis, seminal vesicles, and vas deferens [9]. The inhibition of the enzymes 5α-reductase and aromatase by EDs is one of the main mechanisms responsible for the adverse effects noted, as 5α-reductase is required for the conversion of the androgens to DHT [10], whereas aromatase catalyses the metabolism of androgens to oestrogen [9].

As consequence, EDs affect human reproduction as well as human post and pre-natal development. In fact, infants can be affected already at prenatal level due to maternal exposure to ED (reviewed in [11]). Epidemiological studies have reported an overall decline of male fertility and an increased incidence of diseases or congenital malformations of the male reproductive system [12]. Specifically, it has been observed a decreased sperm count in semen over time which inversely correlates with the incidence of diseases such as testis cancer, cryptorchidism and hypospadias [13]. This trend, known as testis dysgenesis syndrome, was first reported in 1992 by a Danish study that found a 50% decrease in sperm count in the male population across the 1938–1992 period [14]. These reports alarmed both general population and public authorities. In particular, great attention has been given to those chemicals, or their metabolites, that have estrogenic properties or antagonistic effects on the activity of androgen or even inhibiting their production.

These compounds have therefore the potential of interfering with important physiological processes, such as masculinization, morphological development of the urogenital system and secondary sexual traits [15].

The current review discusses the detrimental effects of EDs exposure on male health and fertility, by providing an overview of experimental pre-clinical studies on animal models and humans, when available, and by reporting epidemiological observational studies in humans.

### Phthalates

There are numerous substances with a recognized anti-androgenic effect, from air and ground pollutants to plasticizers. In the latter category, phthalates (Ps) are the most investigated compounds as they are employed in virtually all industrial applications and consumer products as additives. Ps are inexpensive synthetic chemicals and have been widely used as plasticizers in a broad range of industrial and commercial products [16, 17]. The most commonly used phthalates are di-(2-ethylhexyl) phthalate (DEHP), di-n-butyl phthalate (DBP), diethyl phthalate (DEP), and benzylbutyl phthalate (BzBP). More than 75% of DEHP produced worldwide is used in plastic products. The other Ps are largely used in personal care products like foams, shampoos, dyes, lubricants, and food packaging materials [18]. Since these compounds are not covalently-bound polymers, their exposure to heat over time has the potential to favour their migration into food [19]. Human exposure to environmental pollutants from foodstuff poses health risk for the general population. Plasticizers such as phthalate esters, because of their anti-androgen and estrogen-like activity, are indicated as major endocrine disruptors. Both in vitro and in vivo toxicology studies have demonstrated their endocrine disrupting potential in model organisms, with endpoints such as antiandrogen effects, reproductive abnormalities, testicular lesions and reduced sperm production [20] (Fig. 1). However, dose ranges used for traditional reproductive toxicological studies were much higher than those observed in human epidemiological studies. Therefore, it is not surprising that these studies do not entirely align with the human studies. Controversially, in vitro and in vivo toxicology studies with low exposures to Ps were linked to decreased semen quality and male infertility in animals, as well as to decreased androgen production and steroidogenesis [21–30]. Ps have mostly shown the antiandrogen effect on testicular function during steroid formation [31–33]. Several in vitro or in vivo studies also showed that Ps, as well as their metabolites (e.g., DEHP/MEHP, DBP/ MBP) have stimulatory effects at low doses through inducing the production of progesterone, testosterone, steroidogenesis-related proteins and gene expression [23, 24, 26, 27, 29, 30]. The adverse effects of Ps on sperm quality were confirmed by ex vivo studies, where spermatozoa were incubated in vitro and exposed with high concentrations of phthalates [34]. It was reported that the sperm motility was decreased and that cytotoxicity was caused at long-term exposures (> 3 days) of human semen samples to the metabolite DEHP [34]. In parallel DHEP has been shown to inhibit testosterone production, when cultured in vitro with explants derived from human testes [35].

Epidemiological studies report an association between Ps exposure and altered seminal parameters have been reported [36](Table 1). It is important to note that exposure of infants to Ps is mainly due to both maternal exposure and breastfeeding. In fact, breastmilk levels of the phthalate metabolites are positively associated with maternal diet and water consumption. In Korea, breast feed infants exceeded the reference daily dose of DEHP by 8% and of DBP by 6% [37]. More recently, the urinary levels of Ps metabolites were related to infertile biomarkers and infertility in Chinese men [38].

**Table 1:** Summary of epidemiological observational studies on the effects of EDs exposure on semen quality and endocrine function in humans

EDs	Population	Design	Main Findings	Ref.
Ps	168 men from subfertile couples	CC	Decreased sperm motility and concentration	[39]
	463 male partners of subfertile couples	C	Decreased sperm concentration and mobility	[42]
	150 men	C	Decreased sperm concentration	[43]
	379 men from an infertility clinic	CC	Increased DNA damage	[40]
	65 asthenospermic, 65 oligoasthenospermic, 50 fertile males	CC	Decreased sperm motility	[34]
	425 men from an infertility clinic	P	Decreased testosterone, estradiol,and free androgen index	[44]
BPA	42 occupationally exposed and 42 occupationally nonexposed men	CS	Lower FSH in occupationally exposed men. No differences in LH and fT.	[79]
	307 men from general population	CS	No associations with E2, SHBG, and fT. Associated with higher T.	[80]
	167 men attending a fertility clinic	P	Associated with lower inhibin B and LH and higher FSH. No relationship with T, SHBG, E2, fT, T3, T4, and TSH.	[133]
	190 men attending a fertility clinic	CS	Associated with lower sperm concentration, normal morphology and motility. No association with total sperm count. Associated with higher sperm DNA damage.	[74]
	315 fertile men from prenatal clinics	CS	Associated with lower FAI and FAI:LH and higher SHBG. No association with semen parameters, FSH, LH, T, inhibin B, and fT.	[75]
	218 occupationally exposed and nonexposed men	P	Associated with lower sperm concentration, total count, normal motility and vitality in all men. Associated with lower sperm concentration, normal motility and vitality in occupationally exposed men. Associated with lower sperm concentration in occupationally nonexposed men. No association with ejaculate volume and morphology.	[76]
	149 male partners of couples undergoing IVF treatments	P	Associated with lower total sperm count, concentration, and vitality. No association with other semen quality parameters.	[5]
	308 young men from general population	CS	Associated with lower progressive motility. No association with other semen quality parameters. Associated with higher T, LH, E2, and fT. No association with FSH, inhibin B, and SHBG.	[77]
	418 male partners of couples trying to become pregnant	P	Associated with lower % sperm DNA fragmentation. No association with semen quality parameters	[78]
OPs	94 cases and 95 controls	CC	Decreased sperm concentration and motility	[92]
	31 sprayers and 80 controls	CC	Pesticide sprayers had significantly reduced seminal volume, percentage of motility, percentage of sperm with normal morphology, serum LH and T levels, increased time of liquefaction, seminal pH, percentage of immature sperm morphology,	[93]
	32 cases, 46 internal controls, 22 external controls	CC	Sperm motion parameters; sperm progression and beat cross frequency in the exposure group were decreased significantly compared with the internal and the external control groups.	[94]
PFCs	105 men from general population	R	Association of PFOS and PFOA with abnormal sperm morphology; no association between other PFCs and semen parameters or reproductive hormones	[116]
	256 non-exposed adult men attending infertility clinic	CS	No association with semen parameters; positive correlation of serum PFOA and PFOS with LH	[110]
	604 fertile men from general population	C	No association between PFCs and apoptotic markers or reproductive hormones emerged; slight increase in SHBG and DNA fragmentation with increased PFOA exposure	[119]
	588 partners of pregnant women	C	Association between PFOS and abnormal sperm morphology; positive association between PFOA and semen motility	[118]
	247 healthy men from general population	CS	PFOS levels were negatively associated with testosterone; negative association between PFHpS and sperm motility; other PFCs were not significantly associated with semen quality or reproductive hormones	[134]
	169 men from an exposed pregnancy cohort	R	Association between PFOA and reduced sperm concentration and total sperm count; association between PFOA and increased levels of LH and FSH; no association between PFOS and any of the measured parameters	[114]
	501 couples discontinuing contraception	C	Association with abnormal sperm morphology and sperm immaturity for at least 2 PFCs combined	[117]
	59 male patients attending the Centre for Couple Sterility	P	Significant increase in alterations of sperm parameters in PFC-positive subjects; disomy and diploidy rates were significantly increased in PFC-positive males; sperm DNA fragmentation index resulted significantly increased in PFC-positive subjects	[120]
Cd	60 Infertile patients and 40 ferile controls	C	Significant negative correlation was observed between serum Cd level and total sperm count, sperm viability, sperm motility and normal sperm morphology. A positive correlation was also observed between seminal plasma Cd and FSH.	[135]
	140 Infertile patients, 15 Sperm donors and 35 Unselected males	C	The percentage of motile sperm and sperm concentration correlated inversely with seminal plasma cadmium among the infertility patients	[136]
	73 infertile patients and 46 fertile controls	CS	A negative association between seminal cadmium concentration and sperm concentration and sperm motility was found	[129]
	61 infertile patients	CC	There was a significant positive association between the percentage of immotile sperms and seminal plasma levels of cadmium.	[137]
	149 environmentally-exposed males	CS	Significant negative correlation between blood plasma levels of cadmium and normal sperm morphology. No correlation was found between cadmium and other seminal parameters.	[138]
	56 environmentally-exposed males	CS	Significant negative correlation between seminal plasma cadmium levels and total sperm count and sperm concentration. No association was found between cadmium and other seminal parameters	[139]
	219 infertile patients	CS	No significant association was found between cadmium exposure and seminal parameters	[140]
	123 infertile patients	CS	Serum cadmium was significantly associated with a decrease in testis size and an increase in serum estradiol, FSH and testosterone	[141]
	27 occupationally-exposed workers and 45 sperm donors	CS	The concentrations of cadmium did not show any correlation with parameters of semen analysis.	[142]
	1052 men attending fertility clinics	CS	Urinary levels of cadmium were significantly inversely associated with progressive sperm motility and total motility	[143]
	587 men from the general population	CS	Inverse associations between Cd and semen volume, progressive motility and sperm morphology were found across the whole group	[144]

EDs: Endocrine Disruptors; Ps: Phtalathes; BPA: Bisphenol A; Ops: Organophosphate pesticides; PFCs: Perfluoroalkyl Substances; CC: Case-Control study; CS: Cross-Sectional study; C: Cohort study; P: Prospective Study; R: Retrospective study

Studies that were conducted in human populations corroborated the in vitro findings and suggested that exposure to phthalate metabolites is correlated with lower motility of spermatozoa in men from subfertile couples [39]. The DNA damage induced in spermatozoa, the sperm motility and the morphology of the spermatozoa were weakly associated with the exposure to Ps [40–43], whereas with regard to the disruption of the hormonal function, an inverse association between MEHP exposure and testosterone and oestradiol levels was reported [44].

Data available on the effect of Ps on male reproductive health is limited, largely confined to specific cases of infertility [45]. Ps are rapidly metabolized and excreted in urine and feces and therefore the assessment of exposure to Ps in human relies on the measurement of urinary concentrations of phthalate metabolites. However, little or even no attention is given to the possible accumulation of un-metabolized Ps in different tissues [46]. This evidence rises some concerns about the appropriateness of parameters employed as index of exposure to contaminants, in particular for those substances like Ps that, showing specific tissue accumulation, may exert risk associated to long term exposures [32]. To this regard, quantification of both parent compound and corresponding metabolites in specific body fluids may represent an informative parameter with better correlation with clinical parameters [33].

### Bisphenol A

In addition to phthalates, human exposure to the ED Bisphenol A (BPA) affects endocrine-reproductive function in males (Fig. 1). BPA is a high production-volume chemical that is widely used in the manufacture of consumer products such as polycarbonate plastics, epoxy resin liners of canned foods, some dental sealants and composites, and thermal receipts [47]. Due to its widespread use in consumer products, exposure to BPA is ubiquitous. In the United States, more than 90% of urine samples obtained from participants in the 2003–2004 and 2011–2012 National Health and Nutrition Examination Survey (NHANES) had BPA concentrations above the limit of detection [48, 49]. Exposure to BPA has garnered concern and regulatory attention over the past decade owing to its potential endocrine disrupting effects. Specifically, in vitro studies have shown that BPA binds to ERα and ERβ, producing weak estrogenic activity [50, 51]. BPA has also been cited for its ability to bind to the AR, peroxisome proliferator–activated receptor γ, and thyroid hormone receptor in experimental animal studies [52]. For example, doses below the present lowest observed adverse effect level (LOAEL; < 50 mg/kg) for BPA were associated with decreased sperm counts [53–57], impaired sperm motility [53, 55, 56, 58], and increased sperm DNA damage [55, 58–65]. In addition, doses below the present LOAEL for BPA were related to decreased testosterone levels [56, 63, 66–68]. Most animal studies concluded that BPA was a testicular toxicant [69–72]. There are differences across studies related to methodologic aspects such as dose, exposure route, timing, and outcomes measured (reviewed in [73]).

In humans, there is a growing body of literature exploring the associations between male urinary BPA concentrations and semen quality parameters, DNA damage, and reproductive hormones [5, 74–80] (Table 1) and a few studies on paternal urinary BPA concentrations and markers of couple fecundity and fertility such as time to pregnancy and live birth [28, 81]. Only six studies have explored the relationship between urinary BPA concentrations and semen parameters, and two of these studies also examined the association with sperm DNA damage [74, 78]. In the only prospective study to date, Li et al. explored the association of urinary BPA concentrations on semen parameters among 218 factory workers from four regions in China [76]. Their study found a negative association between urinary BPA concentrations and sperm concentration, total sperm count, sperm vitality, and sperm motility. The epidemiologic literature investigating the endocrine disrupting effects of BPA on male reproductive hormones also is limited and presents heterogeneous results. To date, one study has explored this association among men occupationally exposed to BPA [79], two studied the association among men from the general population [77, 80], and two studies investigated this association among either fertile men or subfertile men from a fertility clinic [74, 75]. The association of male urinary BPA concentrations with couple reproductive outcomes was recently assessed in two studies. Using the EARTH study cohort consisting of subfertile couples undergoing fertility treatment at MGH, Dodge et al. examined the associations of paternal urinary BPA concentrations with fertilization, embryo quality, implantation, and live birth among 218 couples who underwent 195 intrauterine inseminations and 211 in vitro fertilization cycles [81]. No associations between paternal urinary BPA concentrations and reproductive outcomes following fertility treatment were found. The association of paternal urinary BPA concentrations with couple reproductive outcomes was also investigated in the LIFE study of 501 couples discontinuing contraception with the intention of becoming pregnant. Similarly to the study among fertility clinic patients, Buck-Louis et al. did not find association between paternal urinary BPA concentrations and time to pregnancy [28], but interestingly higher paternal urinary BPA concentrations were significantly associated with fewer male births. Although the epidemiologic literature on this topic is growing, the evidence supporting an association between urinary BPA concentrations and male reproductive health in humans remains limited and inconclusive (Table 1). Several methodologic differences could explain discrepancies between human studies. First, studies included different study populations, including fertile males who may be less susceptible to the effects of BPA than would sub-fertile men. Second, the distribution of urinary BPA concentrations varied across studies. If there is a nonlinear association between BPA exposure and markers of reproductive health then we may not find consistent results across study populations with markedly different exposure levels. However, it is worth noting that contradictory results were found even among populations with similar urinary concentrations. Moreover, if exposure to BPA is not constant (within-individual variability is known to exist), the time window of BPA exposure captured in cross-sectional studies (e.g., the last 24 h) may not be the biologically relevant exposure window (e.g., the last 90 days for spermatogenesis). Third, all studies measured adult exposure, but none considered early life exposure (e.g., prenatal or peripubertal windows) which may be more sensitive to effects of BPA. Finally, residual confounding factors correlated with BPA exposure and semen quality were not accounted for.

### Organophosphate pesticides

Organophosphate (OPs) pesticides are one of the most widely used class of pesticides for agricultural purposes [82]. They are metabolized by xenobiotic metabolizing enzymes, notably the cytochrome P450 (CYP) and the Paraoxonase (PON) families of enzymes and are therefore not persistent in the environment [83]. The exposure to OPs is assessed by the detection of their corresponding secondary metabolites notably the dialkyl phosphates in biological matrices such as urine [83–85]. The exposure of humans to OPs can be either professional (chemical plant workers, agricultural workers) or environmental (through soil and water contamination). Organochlorine compounds such as DDT and dioxins are not metabolized by the human body and accumulate for a long period of time [8]. In addition, such compounds appear to be a lot more persistent in the environment compared to organophosphorus compounds. Agonistic effects of MTX, an organochlorine pesticide used as an insecticide that was indented to replace DDT, have been reported for the estrogen receptor subtypes ERα and ERβ, whereas an opposite response was noted for the AR [86–88]. Thiophosphates, a class of organophosphorous pesticides, inhibit P450 enzymes namely, CYP3A4 and CYP1A2 that are involved in the metabolism of estrone and testosterone in the liver [89]. At the molecular level, EDs can affect the expression of steroid and sex hormone related enzymes by inducing their corresponding transcription, via binding to nuclear receptors. Notably, OPs and dioxins have been documented to bind with considerable potency to the aryl hydrocarbon receptor (AhR) that induces the expression of CYP1 genes that in turn metabolizes estradiol (E2) to hydroxylated derivatives [90]. Specific adverse effects on the male reproductive system have been reported to occur by organochlorine pesticides, such as endosulfan and DDT due to the disruption of the hypothalamic–pituitary testes axis and the direct interaction with the sex steroid receptors in the target tissue [91] (Fig. 1). The occupational exposure to pesticides increases the risk of morphological abnormalities in the sperm of farm workers that includes a decline in sperm count and a decreased percentage of viable sperms. OP pesticides such as parathion and methyl parathion can decrease the concentration of the sperm by damaging the seminiferous epithelium, while it has been suggested that pesticide exposure affects sex accessory glands that may also reduce the seminal volume [92, 93]. The exposure to pesticides reduces the seminal volume, increases the seminal pH and increases the abnormal sperm head morphology [93]. In addition, Lifeng et al. demonstrated that sperm motility could be affected by a limited number of pyrethroid pesticides, such as fenvalerate [94]. However, most studies were cross-sectional and due to different participation rates and lack of information on time dimension of the cause–effect relationship cannot be supported. Further controlled studies are needed to make sure about the effects of pesticides on male infertility. Although animal studies confirmed an impact of these chemicals on reproductive health, it should be noted that rats are more sensitive to the effects of pesticide exposure in comparison to humans. Moreover, synergistic, and potentiating effects of multiple chemicals are rarely explored in toxicological research. In spite of several studies and laboratory researches, no consistent view exists on the role of chronic pesticide exposure on semen parameters at present [91].

### Perfluoroalkyl compounds

Perfluoroalkyl compounds (PFCs) are a class of organic molecules characterized by fluorinated hydrocarbon chains extensively used in industry and consumer products including oil and water repellents, coatings for cookware, carpets and textiles. PFCs possess unique physical chemical properties due to their amphiphilic structures and their strong carbonfluorine bonds. Therefore, long chain PFCs are non-biodegradable and bioaccumulate in the environment [95, 96]. PFCs have been found in humans and in the global environment and their toxicity, environmental fate, and sources of human exposure have been a major subject of research. Currently 23 PFCs are available, which includes perfluorooctanoic acid (PFOA) and perfluooctane sulfonate (PFOS), which are the predominant forms in human and environmental samples. However, the stability that makes PFCs desirable for commercial use, also entails that they are environmental contaminants due to their resistance to various modes of degradation [97].

Both in vitro and animal studies on PFCs toxicity have shown a detrimental effect of PFOA and PFOS on testicular function, by the alteration of steroidogenic machinery and subsequent defect of spermatogenesis [98–102]. Among the endocrine effects of PFOS in particular, it should be emphasized that this compound can affect the hypothalamic–pituitary axis activity [103, 104] (Fig. 1). It is also able to exert its toxicity at testicular level [105], as reported in rats [103, 106] and in testis models [107]. According to a recent study on male rats [108], high doses of PFOS orally administered for 28 days seem to modify the relative gene and protein receptor expressions of several hormones of the reproductive axis (GnRH, LH, FSH and testosterone) (Fig. 1).

Various PFCs compounds have been found in human serum [109], seminal fluid [110], breast milk [111] and even umbilical cord [112], suggesting a life-long exposure to PFCs in humans, from foetal stages until the adult life. In addition to their persistence, PFOA and PFOS have been shown to induce severe health consequences, such as neonatal mortality, neurotoxicity and immunotoxicity: PFCs act as endocrine disruptors on the foetus and newborns, leading to developmental defects [113]. This has led to strict regulation of PFOA and PFOS use in industrial processes, as the compounds were added to the Annex B of the Stockholm Convention on Persistent Organic Pollutants. In addition to health concerns on the impact of these substances on foetal development, epidemiological studies have focused also on the relationship between PFCs and human fertility, but recent research has focused mostly on female fecundity. In humans, in utero exposure to PFOA was associated later in adult life with lower sperm concentration and total sperm count and with higher levels of luteinizing hormone and follicle-stimulating hormone [114] (Table 1).

Besides the impact of PFCs on the professionally-exposed populations, recent evidence of pollution from chemical industries producing PFCs have emerged also in the general population from at least four different area worldwide: Mid-Ohio valley in the USA, Dordrecht area in Netherlands, Shandong district in China, and Veneto region in Italy. In the latter, the population at risk includes the cities of Vicenza, Padova and Verona, with 350.000–400.000 potentially exposed subjects, in an area of approximately 150 km^2^ and the consequent contamination of water and food [115]. Despite strong evidence pointing towards a negative role of PFCs on male reproductive function, to date few evidence are available on the actual effect of these substances on seminal parameters in men, with conflicting results [110, 116, 117]. Two cross-sectional studies reported negative associations of PFOS, or high PFOA and PFOS combined, with the proportion of morphologically normal spermatozoa in adult men [116, 118]. Furthermore, in a study of men attending an in vitro fertilization clinic, Raymer et al. [110] reported that luteinizing hormone (LH) and free testosterone significantly and positively correlated with plasma levels of PFOA, although PFOA was not associated with semen quality. Conflicting results are reported also for the association between PFCs and sperm DNA quality, although a significant trend is evident for increased DNA fragmentation in exposed men [117, 119, 120]. In infertile males, PFOS levels were higher than fertile counterparts, together with a higher gene expressions of estrogen receptor (ER) α, ERβ and androgen receptor (AR) [121, 122], suggesting that PFCs activity might be linked also to the genetic expression of sex hormones nuclear receptors. With respect to Androgen Receptor (AR), PFOS and PFOA induce a decrease of the protein expression of this receptor in the hypothalamus and pituitary gland as well as in the testis (Reviewed in [123]). These findings clearly suggest an antiandrogenic potential of PFCs and given the growing evidence suggesting a link between AR disruptors and disorders of male health [124, 125], PFCs effect on AR and consequent derangement of hypothalamic-pituitary axis should be a major concern. However it should be noted that there was a lack of consistent results among the investigated outcomes (Table 1). Subtle associations between higher PFOS and lower testosterone or abnormal semen morphology cannot be excluded. In conclusion, in men, there is little evidence of an association between PFAS exposure and semen quality or levels of reproductive hormones. As is the case for many epidemiological studies, causality cannot be definitively established in these studies, largely because of their cross-sectional design. However the consistency of findings in pre-clinical studies strongly suggests a causal relationship for some endpoints. Some effects are similar to associations seen in humans, whereas other effects cannot be extrapolated to humans given differences in toxicokinetics across species of these chemicals.

### Cadmium

Heavy metals have also been recognized as likely inducers of testicular damage and, to this regard, the toxicity of Cadmium (Cd) as environmental contaminant has been known for several decades. Some industrial activities, such as melting and welding of metals, as well as municipal waste incineration are processes that contribute in the release of heavy metals in the environment. Among environmentally exposed population, tobacco smokers are the most exposed subjects, since tobacco leaves accumulate large amounts of Cd, making tobacco smoke the main source of Cd in smokers. Although the mechanisms of testicular toxicity exerted by heavy metals are still under investigation, the permeation through the blood-testis barrier is acknowledged as a fundamental process [126]. Like plasticizers, heavy metals widely employed, in industry as well as in food and dietary supplements [127–129]. Heavy metals can interfere a the different stages of spermatogenesis resulting in either decrease in sperm count or abnormal increase in sperm counts, sperm DNA damage, and impaired sperm motility [130](Fig. 1). Redox active heavy metals are also found to increase the levels of reactive oxygen species, leading to oxidative stress, induction of DNA damage and apoptosis of spermatozoa together with disruption of the blood-testis barrier and further damaging spermatogenesis [131](Fig. 1). Among heavy metals, Cd has been repeatedly proven to induce reproductive toxicity in the male, which has been extensively reviewed elsewhere [132]. Briefly, experimental studies in animal models strongly support the hypothesis that Cd affects male reproductive function, including spermatogenesis and semen quality, as well as endocrine function (Fig. 1). Indeed, Cd induces severe structural damage to testis vascular endothelium, which ultimately results in necrosis of the testis, and impaired spermatogenesis and testis endocrine function, and affects the BTB integrity, which might lead to susceptibility to toxicity and to the development of autoimmunity against germ cells. Moreover, Cd might induce inflammation and apoptosis within the testis, by means of direct effects on inflammation mediators, and on pro-apoptotic and anti-apoptotic factors, and by interfering with signalling pathways of calcium and cyclic AMP. In addition, Cd exerts targeted effects on selected cell populations of the testis, which include direct cytotoxicity and functional impairment of Sertoli and Leydig cells, and oxidative stress in both somatic and germ cells, mainly by means of mimicry mechanisms and interference with antioxidative activity. Moreover, Cd induces epigenetic modifications in Leydig cells and testis of Cd-treated animals, which might potentially determine an impairmentof semen quality, although these changes were not directly linked to reproductive dysfunction. Lastly, Cd treatment determines a direct disturbance of the hypothalamus-pituitary-gonadal axis (Fig. 1), which might determine the impairment of spermatogenesis and endocrine function (reviewed in [132]). Conversely, evidence from clinical studies is less consistent (Table 1). Observational studies in both environmental and occupational exposed males suggest that Cd have a detrimental effect on semen quality, and can alter endocrine function. Nevertheless, some studies failed to identify differences between Cd exposed and non-exposed subjects, probably due to small-sized study populations, and lack of control for potential confounding variables (reviewed in [132]). Therefore, additional well-designed observational studies, as well as further experimental research in humans, are required to eliminate inconsistencies, and to confirm the effects of Cd on human male reproductive function.

## Conclusions

Endocrine disruptors include a class of chemicals that can potentially cause harmful effects to the male and female reproductive systems. In addition to the classical action of EDs that includes the agonism and/or antagonism with hormone and nuclear receptors, the last decade of scientific research has given significant scientific advances in the field of molecular biology that confirmed endocrine disruption by several compounds by interfering with the cell cycle, the apoptotic machinery and the epigenetic regulation of the target cells [8]. However, action mechanisms should not be generally extrapolated since each chemical has different routes to interfere with endocrine activity. Among the EDCs considered in this Review, there is strong experimental evidence of antagonism with hormone nuclear receptors (AR and/or ER) only for heavy metals, in particular Cadmium, whereas weaker evidence is reported for PFCs, BPA and OPs. The modulation of the downstream genes involved in the steroidogenic machinery is another possible target of EDCs (Fig. 1), leading to decreased androgen production and altered spermatogenesis, as reported for Ps, BPA, OPs and to a less extent PFCs.

However, epidemiological studies have shown controversial and inconsistent results (Table 1). This discrepancy can be attributed to several factors that could affect the outcome of the studies, notably to the complexity of the clinical protocols used, the degree of occupational or environmental exposure, the selection of the target group under investigation, the determination of the variables measured and the sample size of the subjects examined. With regard to the male reproductive system, the contribution of geographical and seasonal variation of the semen parameters must be considered. The majority of the epidemiological studies that have examined chemical exposure and the associated semen quality deterioration are cross-sectional. A longitudinal design would be preferable in studies of semen quality. Despite the lack of consistency in the results of so many studies investigating endocrine-disrupting properties of many different classes of chemicals, the overall conclusion points toward a positive association between exposure to EDs and reproductive system.

Despite methodological differences, major concerns are raised by these chemicals, and fertility evaluation is preferred particularly in specific professionally-exposed male workers. In the general population, public health programmes should lead toward a case-by-case investigation of the chemicals depending on environmental (i.e. water, soil and food) pollution. In particular, heavy metals such as Cadmium, pesticides and BPA raise most concerns to male fertility, with strong evidence linking environmental exposure to reduced semen quality parameters (i.e. concentration, total count, viability, motility) and even increased miscarriage rate in females. Evidence of such effects for phthalates and PFCs is less consistent, also due to the relatively recent interest of the scientific community in the effects of these chemicals on male fertility, and for these reasons more studies are clearly needed. Future studies should focus on a uniform system of the investigation of human populations with regard to the exposure to specific EDs and the direct effect on the reproductive system. In addition, the use of advanced molecular biology techniques that are employed to evaluate DNA damage in spermatozoa should be included.
